# Myocardial enzyme profile and short-term prognosis of patients with prolonged myocardial damage after transcatheter aortic valve replacement

**DOI:** 10.5937/jomb0-52087

**Published:** 2025-10-28

**Authors:** Yan Guo, Meili Liu, Jing Li, Ke Han

**Affiliations:** 1 The First Affiliated Hospital of Xi'an Jiaotong University School of Medicine, Department of Cardiovascular Medicine, Xi'an, Shaanxi Province, China

**Keywords:** aortic stenosis, aortic valve replacement, myocardial enzyme profile, short-term prognosis, aortna stenoza, zamena aortnog zaliska, profil miokardnih enzima, kratkoročna prognoza

## Abstract

**Background:**

This work investigated the changes in myocardial enzyme profile (MEP) and short-term prognosis (STP) in patients with myocardial damage (MD) after transcatheter aortic valve replacement (TAVR).

**Methods:**

100 patients receiving TAVR surgery were selected and rolled into an observation group (Obs group, 50 cases) and a control group (Ctrl group, 50 cases) according to postoperative myocardial status. The changes in MEP before and after the TAVR and the STP within 3 days after surgery were compared and analysed.

**Results:**

Creatine Kinase MB (CK-MB) levels were (21.6±8.8) IU/L, (17.2±7.1) IU/L, and (15.2±6.4) IU/L at 12 h, 24 h, and 72 h after TAVR, respectively, in the Obs group; and the cTnT levels were (0.284±0.13) ng/mL, (0.315±0.15) ng/mL, and (0.363±0.22) ng/mL, respectively, at the same time points. The CK-MB and cTnT levels in the Obs group were increased more obviously based on the conditions in the Ctrl group (P&lt;0.05). After surgery, 23 cases of bleeding occurred in the Obs group, significantly more than 8 cases in the Ctrl group (P&lt;0.05). Differences in ultrasonic test results were not obvious (P&gt;0.05).

**Conclusions:**

The MEP of patients after TAVR generally increased, and the increase in the degree of patients with MD was more significant, which may be an indicator to judge the postoperative MD. Patients with postoperative MD had a higher probability of postoperative bleeding.

## Abstract (sr)

**Uvod:**

Ovaj rad ispituje promene profila miokardnih enzima (MEP) i kratkoročnu prognozu (STP) kod pacijenata s oštećenjem miokarda (MD) nakon transkateterske zamene aortnog zaliska (TAVR).

**Metode:**

Odabrano je 100 pacijenata koji su podvrgnuti TAVR operaciji i podeljeni u grupu nad kojom se vrši posmatranje (Obs grupa, 50 slučajeva) i kontrolnu grupu (Ctrl grupa, 50 slučajeva) prema postoperativnom stanju miokarda. Upoređene su i analizirane promene MEP-a pre i posle TAVR-a i STP u roku od 3 dana nakon operacije.

**Rezultati:**

Nivoi kreatin kinaze MB (CK-MB) bili su (21,6±8,8) IU/L, (17,2±7,1) IU/L i (15,2±6,4) IU/L na 12 h, 24 h i 72 h nakon TAVR-a, respektivno, u Obs grupi; a nivoi cTnT su bili (0,284±0,13) ng/mL, (0,315±0,15) ng/mL i (0,363±0,22) ng/mL u istim vremenskim tačkama. Nivoi CK-MB i cTnT u Obs grupi su bili značajnije povećani u odnosu na stanje u Ctrl grupi (P&lt;0,05). Nakon operacije, zabeležena su 23 slučaja krvarenja u Obs grupi, značajno više u poređenju sa 8 slučajeva u Ctrl grupi (P&lt;0,05). Razlike u rezultatima ultrazvučnih testova nisu bile značajne (P&gt;0,05).

**Zaključak:**

MEP pacijenata nakon TAVR-a je generalno bio povećan, a povećanje stepena pacijenata sa MD je bilo značajnije, što može biti pokazatelj za procenu postoperativnog MD. Pacijenti s postoperativnim MD imali su veću verovatnoću za postoperativno krvarenje.

## Introduction

Aortic stenosis (AS) is a common heart valve disease. With the population’s progress in ageing, the number of elderly people in China is gradually increasing, and the incidence of AS is slowly increasing [1]. Recent epidemiological studies have shown that the incidence of aortic stenosis is 0.2% in the age group of 50 to 59 years, rising to 1.3% in the age group of 60 to 69 years, and as high as 9.8% in the age group of 80 to 89. The causes of AS mainly include rheumatism, congenital malformation, and senile degenerative calcification [2]. Patients with symptomatic aortic stenosis who do not undergo surgery typically have an average survival time of 2 to 3 years, and sudden death occurs in 8 to 32 per cent of patients [2] [3]. Medical treatment for aortic stenosis is not effective [3], and aortic valve replacement is an effective treatment for aortic stenosis [4]. Patients with surgical replacement of aortic stenosis usually achieve a normal life expectancy. Still, for elderly patients with poor cardiopulmonary function, more complications, and critically ill patients with aortic stenosis, surgical valve replacement carries a higher risk of surgery and subsequent loss of surgical opportunity. In 2002, the first transcatheter aortic valve replacement (TAVR) procedure achieved in France, ushering in a new era of transcatheter aortic valve replacement, providing new options for aortic valve replacement. Subsequently, TAVR surgery entered a stage of rapid development, and the safety and effectiveness of TAVR in surgical contraindicated, high-risk, and medium-risk patients have been confirmed in several large randomised controlled trials (RCTs) [5] [6] [7].

Postoperative myocardial damage (MD) manifested by elevated myocardial enzyme profile (MEP) is a possible complication of TAVR [8]. Some early small sample studies suggest that perioperative MEP increase of TAVR may adversely affect the early prognosis of patients, and postoperative MEP increase may be a predictor of poor prognosis of patients [8]. A PARTNER sub-study from a large multicenter randomised controlled study also suggested the effect of postoperative MEP on 1-year all-cause mortality [9]. Despite focusing on the same issues, these early studies used different types of MEPs and group boundaries, resulting in a lack of comparability among other studies. Therefore, in 2012, the Valve Academic Research Consortium (VARC) published the second edition of the standardised definition of the postoperative outcome of TAVR (VARC-2) and clarified the meaning of MD as the outcome of TAVR clinical studies for the first time [10]. Perioperative MD in VARC- 2 was defined as a 15-fold increase in Troponin or a CK-MB increase more significant than a 5-fold increase in the upper limit of normal reference. Some subsequent studies grouped using VARC-2 have shown that the MD defined by VARC-2 may be conservative and that different studies have different outcomes regarding the impact on patient outcomes. However, the above studies were mainly carried out based on the aortic stenosis population in foreign countries, and TAVR surgery started relatively late in China, and there is relevant literature [11].

Therefore, patients receiving TAVR surgery were selected in this work to compare and analyse the changes in MEP in the peripheral blood of patients before and after surgery, as well as the short-term postoperative therapeutic effect of patients, to provide a reference for the future clinical application of TAVR surgery.

## Materials and methods

The local ethics committee of the First Affiliated Hospital of Xi’an Jiaotong University approved the study. All experiments followed relevant guidelines and regulations, such as the Declaration of Helsinki. The patients signed the informed consent form and agreed to be published.

### Research objects

This work included 100 patients who received TAVR surgery in our hospital from 2021 to 2022. Patients were rolled into an observation group (Obs group, 50 cases) and a control group (Ctrl group, 50 cases) according to postoperative myocardial status. Criteria for patients to be enrolled were as follows: (1) patients diagnosed with major aortic stenosis and proposed to undergo TAVR surgery; (2) patients with no previous surgery for heart disease; (3) patients who can perform MEP test regularly according to experimental requirements. Patients were excluded according to the criteria below: (1) patients with surgical valve replacement; (2) patients who die during surgery due to force majeure or other factors; and (3) patients who withdrew from the study early for other reasons.

### Methods of assessing MD

According to the VARC-2 guidelines for clinical endpoints after TAVR published by the International Valvular Academic Research Consortium [12], the standard of MD enzymology after surgery is that at least one test result within 72 hours after surgery suggests that troponin has increased by more than 15 times the upper limit of the reference value. Many previous studies have used this standard to discuss the perioperative MD of TAVR. Combined with the normal reference range of MEP given by the clinical laboratory of our hospital, Troponin-T<0.014 ng/mL, CK-MB 24 IU/L.

In this work, perioperative MD of TAVR was defined as follows: I. cTnT 0.21 ng/mL in at least one MEP measurement within the first 72 hours after surgery; II. If the baseline cTnT was elevated before surgery, the increase relative to baseline was 50% if cTnT 0.210 ng/mL was achieved by at least one MEP measurement within the first 72 hours after surgery.

### Methods for detecting MEP

CK-MB used instruments for Roche’s Roche- Modular DP automatic biochemical analyser and Roche-supplied kits. The cTnT was instrumentalised as a Roche-Modular E170 fully automatic biochemical analyser, with kits provided by Roche.

2 mL peripheral venous blood was collected from patients one day before surgery, 12 h, 24 h, and 48 h after surgery, respectively. Detection methods were described as follows. All samples were centrifuged for 3000 r/min; 10 min later, the isolated serum was placed in an EP tube for the test. The detection method was conducted in strict accordance with the reagent instructions. The CK-MB value was determined by immunosuppression, and reagents RI and RII were added to the samples. cTnT value was determined by the electrochemical luminescence method. Biotinized human anti-TNT monoclonal antibody and ruthenium-labeled anti-TNT monoclonal antibody were added to the specimen to form a sandwich complex, and streptavidin-coated particles were added. When the specimens were ready, they would be put into the instrument and tested by special personnel.

### Indicators for evaluating prognosis

The clinical outcomes used in this study were also selected according to VARC-2 guidelines for clinical endpoints after TAVR published by VARC, including:

I. Mortality: mortality from all causes, including cardiac and non-cardiac deaths. The central causes of death included cardiac causes (myocardial infarction, pericardial tamponade, ruptured aortic aneurysm, etc.), deaths caused by non-coronary vascular events (deaths caused by cerebrovascular events, deaths caused by pulmonary embolism, deaths caused by aortic dissection, deaths caused by ruptured aortic aneurysm, etc.), deaths caused by surgical procedures, deaths caused by heart valves, sudden death, and other causes from unknown causes and non-cardiac deaths (trauma, cancer, suicide, etc.).

II. Myocardial infarction (MI): peri-procedural MI refers to myocardial infarction occurring within 72 hours after surgery where the following criteria are met: new ischemic symptoms and/or ischemic signs, including chest pain, dyspnea, ventricular arrhythmia, new heart failure or worsening heart failure, new ST segment changes, new pathological Q wave of two consecutive leads, hemodynamic instability, and new imaging evidence of abnormal cardiac segmental motion; elevated cardiac biomarkers: at least one examination within 72 hours after surgery indicated that CK-MB increased more than 5 times the upper limit of reference value or troponin increased more than 5 times the upper limit of reference value. If the preoperative baseline has been improved, the increase should be more than 50% of the preoperative level. The peak value should exceed the upper limit of the reference value stated above.

III. Stroke: focal neurological dysfunction or generalised neurological dysfunction lasted for more than 24 hours without remission, or focal neurological dysfunction or generalised neurological dysfunction lasted for less than 24 hours, and neurological imaging could provide imaging evidence of a new bleeding focus or a new ischemic focus, or neurological dysfunction leading to death. Disabling stroke is divided into disabling stroke and Non-disabling stroke according to severity.

IV. Bleeding: including life-threatening bleeding, major bleeding, and minor bleeding. The life-threatening bleeding includes: bleeding resulting in death; bleeding of vital organs (intracranial bleeding, intraframe bleeding, etc.); bleeding causing hypovolemic shock or severe hypotension; and Hemoglobin decreased more than 5 g/L, or resulted in more than 4 U dominant bleeding. Major bleeding included: haemoglobin decreased by more than 3 g/L and dominant bleeding with more than 2 units of whole blood/red blood cells transfused. Minor bleeding was defined as any bleeding that attracts the clinician’s attention (for example, hematoma at the puncture site).

V. Vascular complication: includes major vascular complication and minor vascular complication. Severe vascular complications include aortic dissection, aortic tear, aortic ring tear, left ventricular perforation, new apex aneurysms and pseudoaneurysms, fatal, life-threatening bleeding, vascular injuries associated with the approach site of organ ischemia and local nerve function impairment (dissection, stenosis, perforation, tearing, arteriovenous fistula, pseudoaneurysm, hematoma, nerve injury, etc.), distal vascular embolisation that requires surgery, amputation, or irreversible organ damage, etc.

VI. Acute kidney injury (AKIN): according to the AKIN grading standard [13], the diagnosis and classification of serum creatinine elevation within 48 hours should be made.

VII. Valve malpositioning: including valve migration, valve embolisation, and ectopic valve deployment.

VIII. Surgical success: it is the compound endpoint, and all the following conditions should be met: perioperative survival, correct positioning, and release of a single prosthetic valve to the appropriate anatomical position, no valvular patient mismatch, mean aortic cross-valve differential pressure <20 mmHg or peak flow rate <3 m/s, and no moderate or more significant prosthetic valve regurgitation.

IX. Early safety: composite endpoints 30 days after surgery, including ① all-cause death; ② apoplexy; ③ lethal bleeding; ④ grade 2 or above renal impairment; ⑤ coronary artery obstruction; ⑥ serious vascular complications; and ⑦ artificial valve disorders requiring a second surgery.

### Statistical analysis

Excel 2019 was utilised to record and summarise data. SPSS 20.0 severed for data statistics and analysis. Mean±standard deviation (x̄±s) according to measurement data. Group comparison between using single factor analysis of variance (One-way ANOVA), with *P*<0.05 for a statistically significant difference.

## Results

### Patients

**Figure 1:**
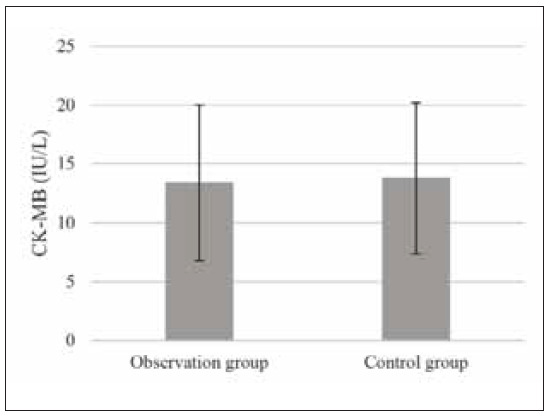
Preoperative CK-MB levels of patients.

**Figure 2:**
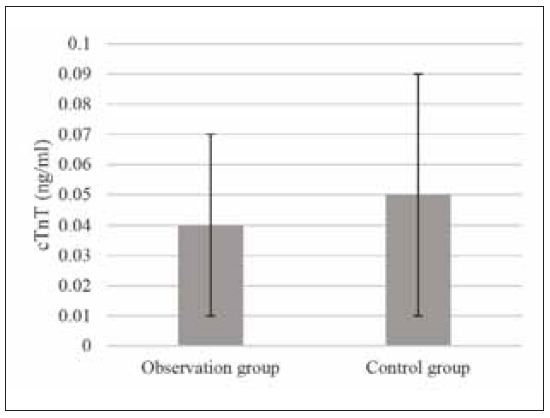
Preoperative cTnT levels of patients

Among the patients enrolled, there were 26 males (52%) and 29 females (48%) in the Ctrl group, and they were (64.2±5.7) years old and with an average body mass index (BMI) of (22.4±2.7) kg/m^2^. Twenty-nine males (58%) and 21 females (42%) were enrolled in the Obs group (62.9±6.4) years old and had a BMI of (22.6±2.3) kg/m^2^. No significant differences were found in all aspects of patients (*P*<0.05) (Table 1). Preoperative MEP test results showed that the CK-MB (13.8±6.4) IU/L and cTnT (0.05±0.04) ng/mL in the Ctrl group exhibited no significant differences with those in the Obs group, which were (13.4±6.6) IU/L and (0.04±0.03) ng/mL, respectively (Figure 1 and Figure 2).

**Table 1:** Baseline data of patients.

	Ctrl group	Obs group	P
Male (cases)	26	29	0.143
Female (cases)	24	21	0.126
Age (years old)	64.2±5.7	62.9±6.4	0.281
BMI (kg/m^2^)	22.4±2.7	22.6±2.3	0.462

### Changes in MEP indexes of patients

**Figure 3:**
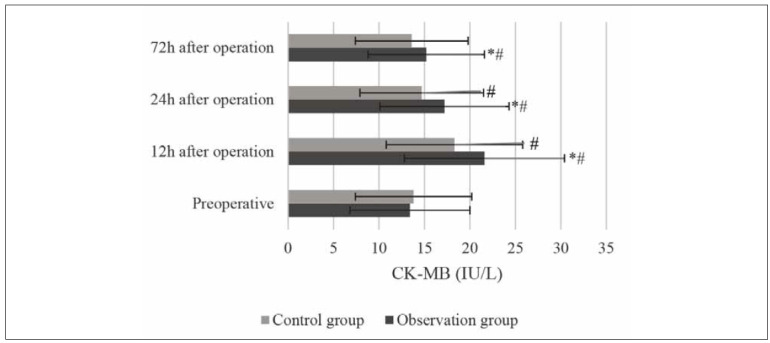
Changes in the CK-MB (* and # indicated significant difference with P<0.05 based on the value in the Ctrl group and before the surgery, respectively).

**Figure 4:**
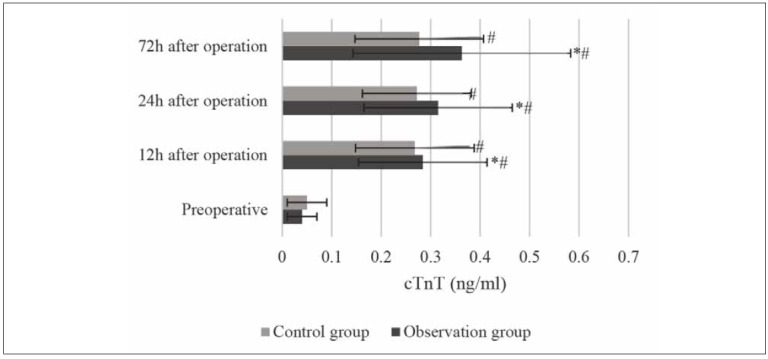
Changes in the cTnT (* and # indicated significant difference with P<0.05 based on the value in the Ctrl group and before the surgery, respectively).

According to the MEP test results, the CK-MB levels of Obs group patients were (21.6±8.8) IU/L, (17.2±7.1) IU/L and (15.2±6.4) IU/L at 12h, 24h, and 72h after surgery, respectively; and the cTnT levels were (0.284±0.13) ng/mL, (0.315±0.15) ng/mL, and (0.363±0.22) ng/mL, respectively at the same time points. In the Ctrl group, the CK-MB levels were (18.3±7.5) IU/L, (14.7±6.8) IU/L, and (13.6±6.2) IU/L at 12h, 24h, and 72h after surgery, respectively, and the cTnT levels were (0.268±0.12) ng/mL, (0.272±0.11) ng/mL, and (0.277±0.13) ng/mL, respectively. The level of CK-MB in both groups showed a downward trend, which in the Ctrl group decreased more obviously (*P*<0.05) (Figure 3). The cTnT level of the patients in both the Ctrl and Obs groups showed a gradual upward trend, with statistical significance (*P*<0.05), and was much higher than the preoperative level (*P*<0.05) (Figure 4).

### Postoperative adverse events of patients

**Figure 5:**
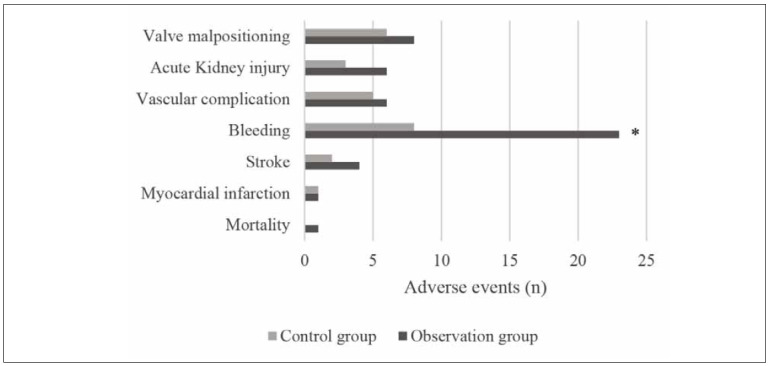
Adverse events of patients (* indicated a significant difference with P<0.05 based on the condition in the Ctrl group).

After surgery, there was 1 case of death, 1 case of myocardial infarction, 4 cases of stroke, 23 cases of bleeding, 6 cases of vascular complications, 6 cases of acute kidney injury, and 8 cases of abnormal position of artificial valves in the Obs group. In the Ctrl group, there was 1 case of myocardial infarction, 2 cases of stroke, 8 cases of bleeding, 5 cases of vascular complications, 3 cases of acute kidney injury, and 6 cases of the abnormal position of the artificial valve. The comparison revealed that the postoperative number of bleeding patients in the Obs group was more than that in the Ctrl group and exhibited a significant difference (*P*<0.05) (Figure 5).

### Postoperative ultrasonic test results of patients

**Figure 6:**
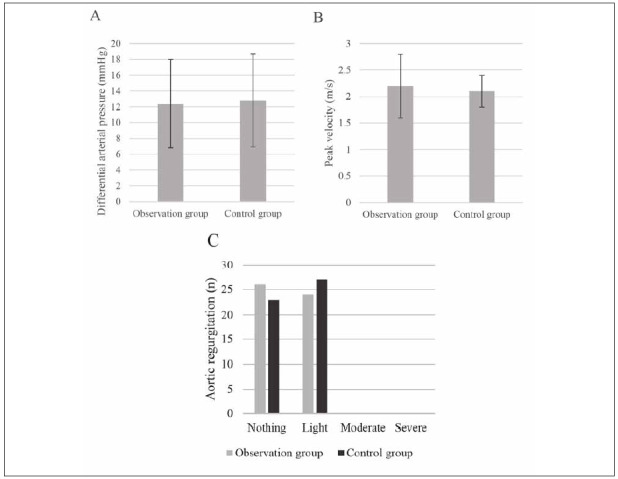
Ultrasound-related indexes of patients.

Postoperative ultrasonic examination results showed that the mean aortic valve pulse pressure was (12.4±5.6) mmHg, the mean peak aortic valve flow rate was (2.2±0.6) m/s, 26 cases had mild aortic regurgitate, and the rest of the patients had no regurgitate. In the Ctrl group, the mean aortic pulse pressure difference was (12.8±5.9) mmHg, the mean peak aortic flow rate was (2.1±0.3) m/s, 23 patients had mild aortic regurgitation, and the rest patients had no regurgitation. No obvious difference was observed in all items (*P*>0.05), (Figure 6).

## Discussion

In this work, MEP-related indicators in MD patients were investigated in detail after TAVR surgery, and the STP of patients was analysed. The ageing of the population increases the prevalence rate of aortic valve disease gradually, and more and more patients with AS have no symptoms or mild symptoms at the early stage. However, when AS develops to severe, the median survival time is about 23 months, and the median survival time can be reduced to 11 months after the onset of heart failure symptoms, so early surgical treatment is the key [14]. As a means of interventional therapy with less trauma and faster recovery, TAVR is an effective treatment, especially for elderly patients with AS, with significant advantages in improving symptoms and reducing mortality [15]. Both early and recent studies have found that increased MEP after TAVR surgery is common, and different researchers agree that increased MEP after TAVR surgery represents a certain degree of MD. However, the MEP threshold required by the definition of MD differs in different studies, resulting in the incidence of MD reported by other studies not being consistent [16] [17]. Until a new set of clinical outcome definitions for TAVR studies were added to VARC-2 guidelines, the criteria for myocardial enzymology were defined as postoperative cTnT increase exceeding 15 times the upper limit of normal reference value or postoperative CK-MB increase exceeding 5 times the upper limit of standard reference value.

The results suggested no significant difference in CK-MB and cTnT between patients with MD and those without MD before surgery; however, after surgery, CK-MB and cTnT increased to varying degrees in the two groups of patients, and the level of CK-MB and cTnT increased more significantly in patients with MD (*P*<0.05). The study of Jo et al. [18] showed that CK-MB and cTnT were more sensitive to the MD of surgical trauma than ischemic injury in patients undergoing heart surgery. In the study of Yang et al. [19], the levels of myocardial enzyme markers in asphyxia newborns were significantly higher than those in asphyxia newborns without MD (*P*<0.05). All of them are consistent with the results of our study. In LTP, MD patients were more prone to bleeding after surgery, but there was no significant difference in other aspects. Sun et al. [20] also found in percutaneous coronary intervention that cTnT or CK-MB levels may be helpful to cardiac biomarkers for monitoring MD, especially 24 hours after surgery, but appear not to affect prognosis.

In conclusion, this work showed that MEP indexes of patients with TAVR after surgery generally increased. MEP indexes of patients with MD increased more significantly, which may be used to judge patients with MD after surgery. The postoperative bleeding rate was higher in patients with MD. However, it was not clear whether the MEP index had a direct relationship with postoperative adverse events. In addition, the MEP test method may have some errors, and the sample size was small, so it was necessary to increase the sample size for a more comprehensive study.

## Dodatak

### Data availability statement

The original contributions presented in the study are included in the article.

### Funding

Not applicable.

### Authors contribution

YG, ML, JL and KH contributed to the design of the study and data collection, performed the data analysis and wrote the manuscript. All authors read and approve the final manuscript version.

### Acknowledgements

Not applicable.

### Conflict of interest statement

All the authors declare that they have no conflict of interest in this work.
